# Impact of Prebiotic on Viability of *Lactiplantibacillus plantarum* D-2 by Encapsulation through Spray Drying and Its Commercialization Potential

**DOI:** 10.4014/jmb.2401.01019

**Published:** 2024-03-29

**Authors:** Changheon Lee, Daeung Yu

**Affiliations:** 1 Interdisciplinary Program in Senior Human-Ecology, Major in Food and Nutrition, Changwon National University, Changwon 51140, Republic of Korea; 2 Department of Food and Nutrition, Changwon National University, Changwon 51140, Republic of Korea

**Keywords:** Probiotic, encapsulation, prebiotic, inulin, viability

## Abstract

This study investigated the impact of inulin (INL) on viability of *L. plantarum* D-2 (LPD2) by encapsulation through spray drying (SD) and its commercialization potential to alternative of conventional wall material maltodextrin (MD). LPD2, derived from sea tangle (*Saccharina japonica*) kimchi, is probiotics exhibiting significant attributes like cholesterol reduction, antioxidant properties, and resilience to acidic and bile environments. To enhance storage viability and stability of LPD2, encapsulation was applied by SD technology. The optimum encapsulation condition with MD was 10% MD concentration (MD10) and inlet temperature (96°C). The optimum concentration ratio of MD and INL was 7:3 (INL3) for alternative of MD with similar encapsulation yield and viability of LPD2. Viability of LPD2 with INL3 exhibited almost 8% higher than that with MD10 after 50 days storage at 25°C. Physicochemical characteristics of the encapsulated LPD2 (ELPD2) with MD10 and INL3 had no significant different between flowability and morphology. But, ELPD2 with INL3 had lower water solubility and higher water absorption resulting in extension of viability of LPD2 compared to that with MD10. The comprehensive study results showed that there was no significant difference in the encapsulation yield and physicochemical properties between ELPD2 with MD10 and INL3, except of water solubility index (WSI) and water absorption index (WAI). INL have the potential to substitute of MD as a commercial wall material with prebiotic functionality to enhance the viability of LPD2 by encapsulation.

## Introduction

Probiotics are beneficial microorganisms that positively impact gut health, skin, oral cavity, and genitals, pioneered by Elie Metchnikoff in the early 1900s [1, 2]. The probiotics market is poised to grow from $46.54 billion in 2017 to $76.85 billion by 2023 [3]. This expansion is driven by heightened consumer awareness of the health benefits associated with probiotics [4].

*Lactiplantibacillus plantarum* D-2 (LPD2) is one of beneficial micremfdjrkdkanoorganisms isolated and identified from sea tangle (*Saccharina japonica*) kimchi, a traditional Korean fermented food [5]. It is known for its ability of withstand acidic and bile environments, making it a suitable probiotic supplement. Additionally, LPD2 possesses cholesterol-lowering and antioxidant properties, contributing to its overall health benefits [5].

Prebiotics are non-digestible substances that selectively promote the growth of beneficial bacteria in the gut, enhancing intestinal health [6]. Inulin (INL), a polymer of fructose, is found in various plants like garlic, onion, and chicory root. It serves as a functional food component with applications as a prebiotic, viscosity modifier, fat or sugar substitute, and dietary fiber [7-9]. INL can improve viability and period of probiotics by preventing heat and dehydration damages during encapsulation and storage, so related researches are actively being conducted [3, 6].

Encapsulation is a technique that employs physical or chemical methods to enclose active components in food through wall materials, preserving their quality during processing and storage [10]. Various encapsulation methods, including freeze, spray, and spray-freeze drying, have been utilized in the food industry to encapsulate probiotics, drugs, and functional food ingredients. Spray drying (SD), an encapsulation technology, involves spraying liquid droplets of a polymer (wall material) and probiotic mixture into a drier, where they come into contact with heated air to form tiny solid particles [11]. SD offers several advantages for probiotic encapsulation, including: control over particle size, continuous production, and rapid drying time [12-14].

Response surface methodology (RSM) is a statistical technique tool for optimizing multiple trial characteristics and identifying the ideal combination of parameters for predicting a specific response to fit experimental data into a theoretical model. The most commonly used and widely applied RSM designs are central composite (CCD) [15, 16].

Physicochemical characteristics (density, flowability, cohesiveness, and structure) of encapsulated powders are crucially influence various properties affecting the commercialization including environmental sensitivity, transportation and deposition behavior, load-bearing capacity, and chemical reactivity [11, 15, 17, 18].

This study aimed to impact of prebiotic (INL) on viability of *L. plantarum* D-2 by encapsulation through SD and its commercialization potential to alternate conventional wall material maltodextrin (MD).

## Materials and Methods

### Materials

*L. plantarum* D-2 was donated from food microbiology lab of Pukyong National University (Busan, Republic of Korea). For the preculture and identify of LPD2, deMan Rogosa Sharpe (MRS) broth (Difco, USA), MRS agar (Difco), and a genomic DNA kit (Chelex bead; Bio-Rad, USA) were utilized. As encapsulation wall materials for encapsulation of LPD2, MD (Max-1000; Matsutani Korea, Republic of Korea) and INL (Orafti-HP; BENEO, Belgium) with over 23 degree of polymerization (DP) were used.

### Identify and Preculture of *L. plantarum* D-2

To identify of LPD2 strain was through a genomic DNA kit, followed by 16S rRNA sequencing employing 27F and 1492R primers. Identification was achieved by comparing the obtained sequence to the National Center for Biotechnology Information (NCBI) BLAST database (https://blast.ncbi.nlm.nih.gov, data not shown). To prepare a preculture of LPD2, 1 ml of the stored stock solution was added to 500 ml of MRS broth and incubated in a shaking incubator (DS 301-FL, Dasol Science, Republic of Korea) at 37°C for 18 h. This incubation time allows the bacteria to grow and reach an initial concentration of 9 log CFU/ml.

### Optimization of Encapsulation Condition of LPD2

MD concentration (X_1_, 10-30%) and SD inlet temperature (X_2_, 80-110°C) and encapsulation yield (Y_1_) and viability of LPD2 (Y_2_) were designated as independent and dependent variables to optimize the encapsulation condition of LPD2. Table 1 presents the coded and decoded values of the independent and dependent variables for CCD design. The relationship between the independent and dependent variables was explored by fitting the dependent variable to a second-order polynomial model Eq. (1).


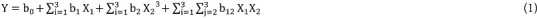


where, Y: Dependent variable, b_0_,b_1_, b_2_, b_12_: Intercept coefficient, X_1_, X_2_ : Independent variables

The absolute residual error (ARE, %) was calculated through the predicted and actual values according to Eq. (2) and expressed as a percentage.

$$\text{ARE(\%) = }\frac{\text{Predicted value - Actual value}}{\text{Actual value}}\times 100$$ (2)

A precultured LPD2 solution (300 mL) was mixed with different concentrations of MD as followed the designed RSM conditions through a magnetic stirrer (HS15-26P, Misung Scientific Co., Ltd., Republic of Korea). The SD process was carried through the spray dryer (ADL311S, Yamato Scientific Co., Ltd., Japan) with fixed parameter (injection rate of 100 ml/h and atomizer pressure of 120 kPa) found from preliminary experiments showing the optimum SD yield.

The encapsulation yield (%) was calculated as the ratio between the weight of the final powder obtained after SD and the total solid content of the feeding solution. The total solid content was the sum of the weight of the pre-cultured solution dried at 105°C overnight and the weight of the added wall material. This calculation was expressed by Eq. (3).

$$\text{Encapsulation Yield (\%) = }\frac{\text{Weights of powder (g)}}{\text{Solid contents of feeding solution (g)}}\times 100$$ (3)

Different concentration ratios of wall materials (MD and INL) were shown in Table 2 for a study of alternative of MD to encapsulate LPD2. Total concentration of wall materials and inlet temperature of SD were fixed based on the result of encapsulation of LPD2 with MD. 300 ml of precultured LPD2 solution was mixed with different concentrations ratio of MD and INL by a magnetic stirrer and then, spray dried. To determine the viability of LPD2 before and after the encapsulation, 1 g of encapsulated LPD2 (ELPD2) was diluted 10-fold with 9 ml of sterile saline solution containing 0.85% NaCl (w/v). 1 ml of the suspension was then transferred to a sterile petri plate and spread onto MRS agar. The petri plate was then incubated at 37°C for 48 h. The viability of LPD2 was calculated through Eq. (4) and expressed as a percentage.

$$\text{Viability (\%) = }\frac{N}{N_{0}}\times 100$$ (4)

N = Number of LPD2 after encapsulation (log CFU/g)

N_0_ = Number of LPD2 before encapsulation (log CFU/ml)

In vitro digestion of ELDP2 was evaluated according to the method by Minekus *et al*. [19]. ELDP2 was exposed to simulated gastric fluid (SGF) for 2 h. Then, it was exposed to simulating small intestinal fluid (SIF) for another 2 h. After digestion, 1 ml of sample was poured with MRS agar and incubated at 37°C for 48 h. The viability of LPD2 was calculated through Eq. (4) and expressed as a percentage. The viability and in vitro digestion of ELPD2 during storage was evaluated at two storage temperatures (4°C-refrigeration temperature and 25°C- room temperature) for 50 day storage.

### Physicochemical Characteristics of ELPD2

The bulk density (ρB) and tap density (ρt) of ELPD2 were determined through the method described by Reddy *et al*. [20]. For ρB, a mass cylinder with a volume of 10 ml was filled with 1 g of ELPD2 without tapping and calculated through Eq. (5). Subsequently, for ρt, the same sample in the mass cylinder was manually tapped by moving it up and down with a vertical distance of 14 mm ± 2 mm and calculated through Eq. (6).

$$\text{ρB=}\frac{\text{Weights of powder (g)}}{\text{Bulk of powdered volume (ml)}}$$ (5)

$$\text{ρt=}\frac{\text{Weights of powder (g)}}{\text{Tapped of powdered volume (ml)}}$$ (6)

The flowability and cohesiveness of ELPD2 were determined through the Hausner ratio (HR) and Carr's index (CI) values. These parameters were calculated based on ρB and ρt of the samples through Eq. (7) and (8) [20].

$$\text{HR=}\frac{\text{ρt}}{\text{ρB}}$$ (7)

$$\text{CI = }\frac{\text{(ρt-ρB)}}{\text{ρt}}\times 100$$ (8)

The water solubility index (WSI) and water absorption index (WAI) of ELPD2 were determined following the method described by Ahmed, Akter, and Eun [21]. For WSI, 1 g of ELPD2 was mixed with 12 ml of distilled water in a 50 ml centrifuge tube. The mixture was then centrifuged at 2,090 ×*g* for 15 min through a centrifuge (Combi R515, Hanil Scientific Inc., Republic of Korea). The supernatant was collected in a pre-weighed aluminum dish, which was subsequently dried in an oven at 105°C overnight. The weight of the dried residue was measured and calculated through Eq. (9). For WAI, the precipitate, the solid particles that settled at the bottom of the centrifuge tube, was also weighed and calculate through Eq. (10) with weight ratio of the precipitate to ELPD2.

$$\text{WSI(\%) = }\frac{\text{Weights of dissolved solids (g)}}{\text{Weight of sample before dissolution (g)}}\times 100$$ (9)

$$\text{WAI (\%) = }\frac{\text{Weights of preipitate (g)}}{\text{Weight of sample before dissolution (g)}}\times 100$$ (10)

The surface morphology of ELPD2 was examined through a scanning electron microscope (SEM, CZ/MIRA I, LHM, Tescan, Czech). Firstly, ELPD2 were subjected to a thin coating of gold ions to enhance their conductivity and prevent charging during SEM analysis. The coated samples were then placed in the SEM chamber, and images were acquired at an accelerating voltage of 3.0 kV and magnifications ranging from 300 to 1,000 times.

### Statistical Analysis

The statistical significance, lack of fit, and the regression coefficient of the RSM were evaluated through analysis of variance (ANOVA) within MINITAB 19 software (MINITAB Ver. 19, MINITAB, USA) To identify statistically significant differences between mean values within each analysis, Duncan's test was employed. This analysis was conducted through SPSS software version 27 (Statistical Package Inc., USA).

## Results

### Optimization of Encapsulation Condition of LPD2

Table 3 shows the influence of MD concentration (X_1_, 10-30% w/v) and SD inlet temperature (X_2_, 80-110°C) on encapsulation yield (Y_1_) and viability (Y_2_) of LPD2 after encapsulation through SD. The actual values of Y_1_ and Y_2_ ranged from 70.73 ± 0.22% and 93.31 ± 0.35% to 79.15 ± 1.18% and 98.01 ± 0.24 %, respectively. The actual values of Y_1_ decreased from 79.15 ± 1.18 to 70.73 ± 0.22% with increasing of X_1_ from 10 to 30%. And, Y_2_ decreased from 98.01 ± 0.24 to 93.31 ± 0.35% with increasing of X_1_ and X_2_ from 10% and 80°C to 30% and 110°C, respectively. The optimum condition for encapsulation of LPD2 with MD were identified through response surface analysis by least-squares regression. Table 4 presented the ANOVA results regarding the parameters and response model equation and the optimum condition for encapsulation of LPD2. To validate the reliability of the models, assessments were conducted for lack of fit and R^2^ values. For Y_1_ and Y_2_, the R^2^ values were 0.8366 and 0.7465 with 0.180 and 0.002 of lack of fit in the response model equations, respectively. The R^2^ value over 0.7 and a lack of fit *p*-value exceeding 0.05 signify the most suitable model equation. Consequently, the derived response model equations were considered suitable for optimizing the encapsulation conditions of LPD2 with MD through SD. The optimum condition was determined to be 10% and 95°C for X_1_ and X_2_, respectively. Based on the optimum X_1_ and X_2_, the predicted and actual values of Y_1_ and Y_2_ were 79.15% and 75.34 ± 1.38% and 97.14% and 92.18 ± 0.48%, respectively. The AREs for the Y_1_ and Y_2_ were 5.05% and 5.38%, respectively.

### Encapsulation of LPD2 with INL

Based on the results of ELPD2 with MD, X_1_ (10%) and X_2_ (95°C) were fixed. The impact of INL on encapsulation yield and viability of LPD2 were shown at Table 5. Encapsulation yields and viability of ELPD2 ranged from 74.73± 0.62% and 90.08 ± 0.58% to 76.69 ± 1.64% and to 98.80 ± 0.50%, respectively. Encapsulation yields depending on the various mixing rations of MD and INL had no significant difference. But the viability of encapsulation for LDP2 of various mixing ratios with MD and INL was ranged from 90.08 ± 0.58% to 98.80 ± 0.50%. The viability of LPD2 decreased with increasing concentration of INL. However, there was no significant difference of the viability of LPD2 after encapsulation with between MD10 and INL3.

### Storage Stability of ELPD2

**Fig. 1:**
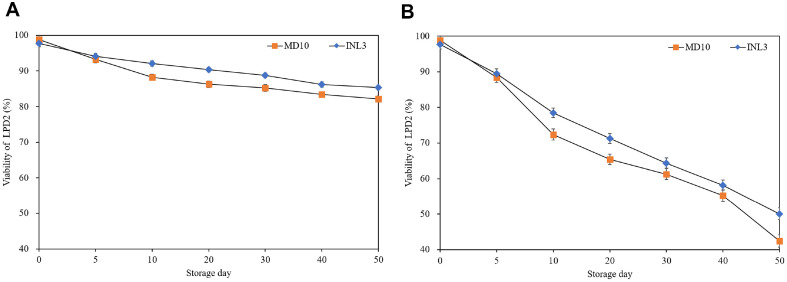
Viability of LPD2 after encapsulation with MD10 (■) and INL3 (◆) during 50 days storage at 4°C (A) and 25°C (B).

Fig. 1 shows the viability of LPD2 after encapsulation with MD10 and INL3 during 50 days storage at 4°C and 25°C. At 4°C, ELPD2 with MD10 and INL3 ranged from 97.70% and 98.80% to 82.18% and 85.38%, respectively. While at 25°C, ELPD2 varied from 97.70% and 98.80% to 49.35% and 54.38%, respectively. Irrespective of wall materials, the viability of LPD2 decreased with increasing storage temperature and day. After a 50 days storage, the viability of LPD2 encapsulated with MD10 and INL3 decreased 16.82% and 7.34 % at 4°C and 50.05% and 42.24%at 25°C, respectively. After a 50 days storage, the viability of LPD2 at 4°C exhibited approximately two times higher than that at 25°C. Especially, the viability of LPD2 with INL3 was almost 8% higher than that with MD10 after 50 days storage at 25°C. The enhanced viability of INL3 in comparison to MD10 during storage shows the prebiotic effect of INL.

**Fig. 2:**
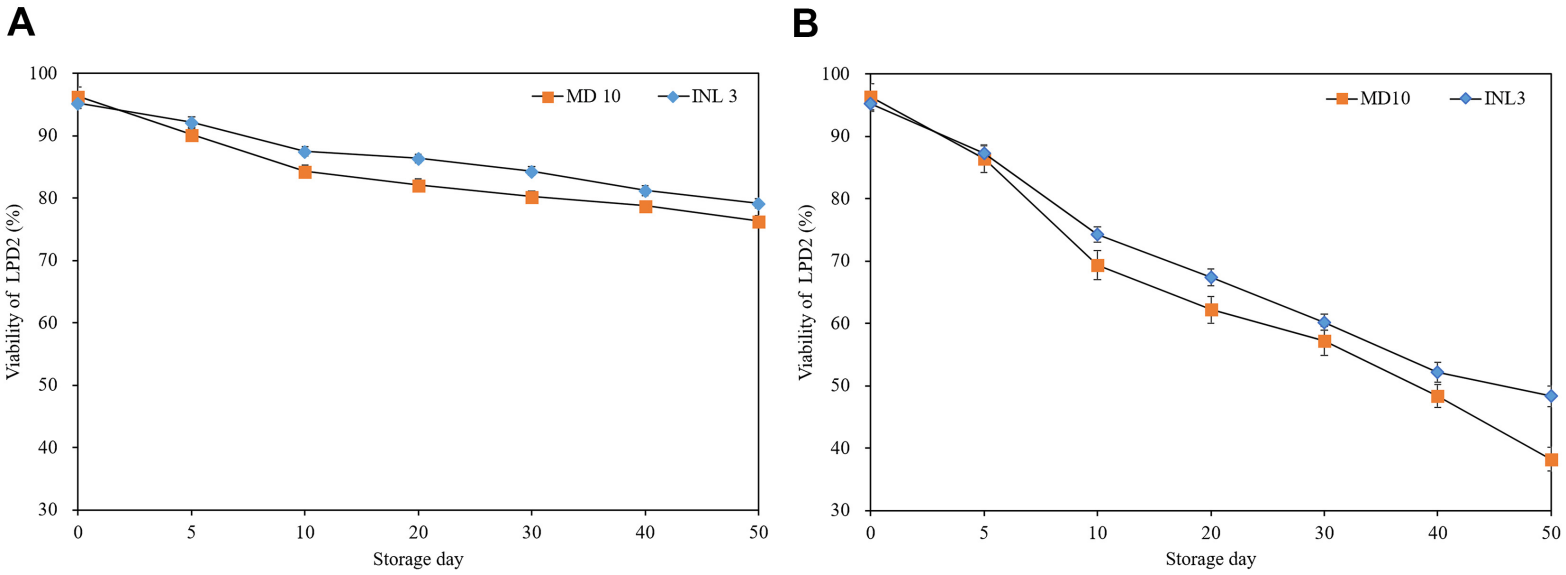
Viability of encapsulated LPD2 after in vitro digestion with MD10 (■) and INL3 (◆) during 50 days storage at 4°C (A) and 25°C (B).

Fig. 2 shows the viability of ELPD2 with MD10 and INL3 after in vitro digestion were investigated during 50 days storage at 4°C and 25°C. At 4°C, ELPD2 with MD10 and INL3 ranged from 95.28% and 96.38 % to 76.38% and 79.18% at 4°C, respectively. While at 25°C, ELPD2 varied from 95.28% and 96.38% to 38.24% and 48.32%, respectively. Irrespective of wall materials, the viability of LPD2 decreased with increasing storage temperature and day. After a 50 days storage, the viability of LPD2 encapsulated with MD10 and INL3 decreased 20.00% and 16.10% at 4°C and 58.14% and 46.96% at 25°C, respectively. After a 50 days storage, the viability after in vitro digestion of LPD2 at 4°C exhibited approximately two times higher than that at 25°C. Especially, the viability of LPD2 with INL3 was almost 10% higher than that with MD10 after 50days storage at 25°C. High loss in relative viability of ELPD2 after in vitro digestion with MD10 was observed compared to INL3. It indicated that INL had high protection ability for LPD2 during in vitro digestion due to a low water solubility.

### Physicochemical Characteristics of ELPD2

**Fig. 3:**
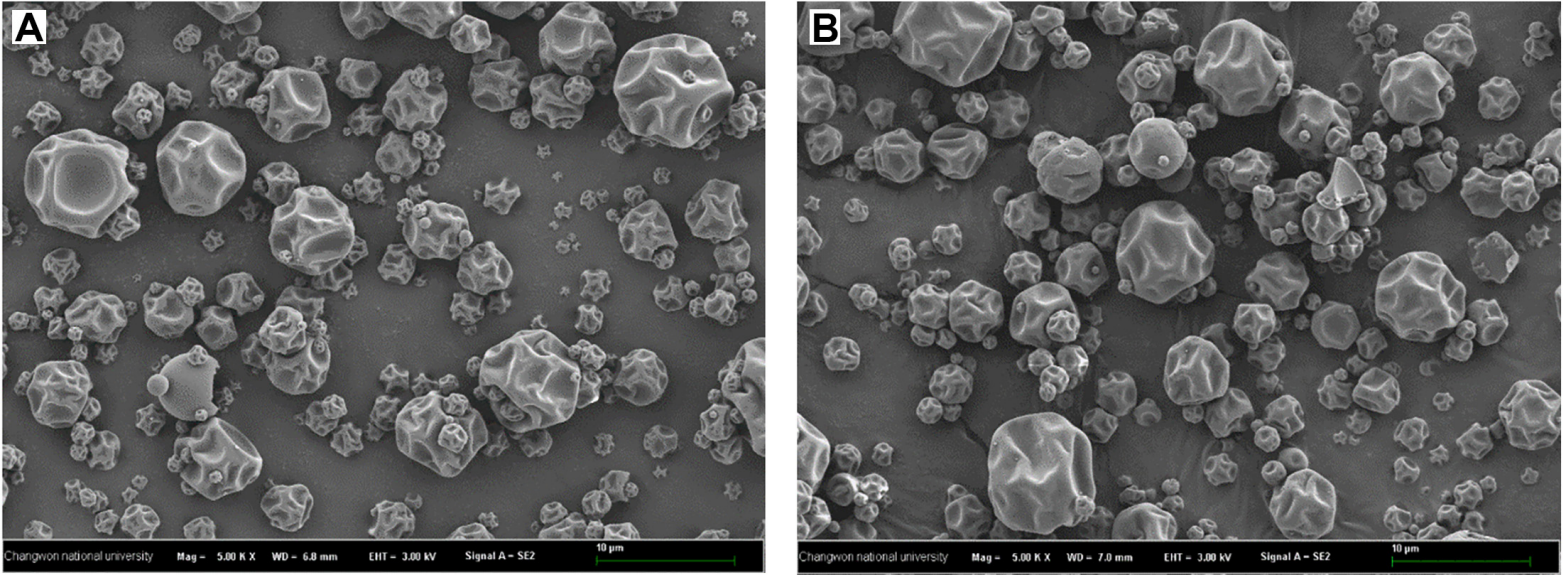
SEM images of encapsulated LPD2 with (A) MD10 and (B) INL3 at 500x magnification.

In Table 6, the physicochemical characteristics of ELPD2 with MD10 and INL3 were presented. The ρB and ρt of ELPD2 with MD10 and INL3 were evaluated as 0.47 ± 0.01 and 0.59± 0.04 and 0.63 ± 0.08 and 0.72 ± 0.05, respectively. The HR and CI values of ELPD2 with MD10 and INL3 were estimated as 1.26 ± 0.12 and 1.32 ± 0.03 and 24.59% ± 1.72 and 24.68 ± 1.80% respectively. There was no significant differences in HR and CI values. The WSI and WAI values for ELPD2 with MD10 and INL3 were measured as 57.08 ± 3.48% and 66.23 ± 4.90% and 0.63± 0.04 and 0.68 ± 0.06, respectively. The Fig. 3 shows SEM images of ELPD2 with MD10 and INL3. The surfaces of the ELPD2 had smooth and free of any cracks or breaks regardless of wall materials. The ELPD2 with INL3 had larger size and smoother surface compared to that with MD10.

## Discussion

### Optimization of Encapsulation Condition of LPD2

Usually increasing wall material concentration led to decreasing encapsulation yield due to enhanced viscosity causing increased adhesion to the SD chamber wall [22, 23]. Lian *et al*. reported reduced encapsulation yield and viability of *B. longum* B6 and *infantis* with gelatin, gum arabic, and soluble starch concentrations over 30% [24]. The decreased in viability was attributed to cellular damage, including DNA and RNA denaturation, cytoplasmic membrane dehydration, and cell membrane rupture by water loss from high inlet temperatures during SD [16, 23]. Bustamante *et al*. reported encapsulation of *L. acidophilus* with polysaccharides (inulin, mucilage of chia, and flaxseeds) through SD, the highest viability was observed at the lowest inlet temperature (90°C) [3]. Kim *et al*. reported that ARE below 10% indicated the most fitting response model equation [25]. Therefore, the derived response model equations were considered suitable for optimizing the encapsulation condition of LPD2 with MD through SD.

### Encapsulation of LPD2 with INL

Decreasing viability of LPD2 with increasing concentration of INL was attributed to types of INL dividing into two categories based on the DP [8, 26]. Low DP (≤ 23) was consisted of short chains of linked fructose units and possessing prebiotic functions, while high DP (>23) serve as stabilize structure of encapsulated powder [26]. Canbulat and Ozcan reported that INL with high DP reduced the viability of *L. rhamnosus* in yogurt more than that with low DP [7]. However, other researchers reported that INL with high DP can increase the storage stability of powder and improve SD yield [7]. Therefore, encapsulation of LPD2 with INL3 was the suitable for improving viability of LPD2 without the loss of encapsulation yield compared to that with MD10.

### Storage Stability of ELPD2

The viability of LPD2 after encapsulation during storage decreased with the increasing storage period and temperature resulted from increased metabolic activities of encapsulated probiotics by increasing storage period and temperature. After a 50 days storage, the viability of LPD2 after encapsulation at 4°C exhibited approximately two times higher than that at 25°C. Previous research has demonstrated that elevated storage temperatures led dehydration resulting in cellular damage and diminished metabolic activity reduced impact probiotic viability [27, 28]. And Desmond *et al*. demonstrated high viability of spray-dried *L. paracasei* with acacia gum at lower temperatures (4°C and 15°C) compared to storage at 37°over 8 weeks [10]. Especially, the viability of LPD2 with INL3 was almost 8% higher than that with MD10 after 50days storage at 25°C. It was attributed to the chemical structure of MD with short chain of polysaccharides resulting in elevating hygroscopicity causing disruption in the stability of LPD2 during storage [29]. In contrast, INL exhibited significantly lower solubility compared to MD due to its high DP [30]. Bustamante *et al*. reported that viability of *L. acidophilus* 05 after encapsulation with polysaccharides (inulin, mucilage of chia, and flax seeds) was higher stored at 4°C in comparison to that at 25°C after a 50 days storage. And, also reported of high viability after in vitro digestion using INL as wall material. It was due to the slow solubility of INL after used as a wall material for encapsulation [3]. INL relatively had a low solubility which could delay rehydration of encapsulated probiotics thereby delaying the release of probiotics into gastrointestinal tract [31].

### Physicochemical Characteristics of ELPD2

The results showed that there was no significant difference in the ρB and ρt of ELPD2 with MD10 and INL3 resulting in no differences of between HR and CI values indicating similar high flowability and low cohesiveness (easy handling and storage) of ELPD2 with both wall materials [15, 32]. These make a better commercialization characteristics of ELPD2. MD, having an abundance of water-attracting sites, exhibited greater solubility compared to INL resulting in enhancing hygroscopicity and solubility [32]. So, WSI of ELPD2 with MD was higher than that of ELPD2 with INL3. Hygroscopicity and solubility of apple pulp and sweet potato powders after encapsulation by SD was increased with increasing MD concentration as a wall materials [21, 32]. Other researcher reported that INL had very low solubility compared to MD due to the linked polymer chains of INL structure. In the point of view of probiotics powder, low WSI and high WAI are preferable for viability of probiotics during storage. Therefore, INL3 had a potential to alternate conventional wall material (MD) with prebiotic functionality to enhance the viability of LPD2 by encapsulation. The SEM images of the ELPD2 had a rough and spherical surface, which was due to the shrinkage of the particles as the moisture evaporates from the feeding fluid droplet. Smoother and more spherical surfaces observed in ELPD2 with INL3 was attributed to the complex blend of branching polysaccharides of INL3, known for its capacity to create dense and uniform particles.^30^ So, ELPD2 with INL3 are more stable, flow more easily, and release their contents more slowly compared to ELPD2 with MD10.

## Conclusion

In conclusion, our results suggest that INL had a potential to alternate conventional wall material (MD) with prebiotic functionality to enhance the viability of LPD2 by encapsulation. The study was aimed that impact of prebiotic (INL) on viability of LPD2 by encapsulation through SD and its commercialization potential to alternative of conventional wall material (MD). Further research should be conducted on alternative of conventional wall materials by finding the optimum mixing ratio of functional prebiotics (tragacanth gum, flexed gum, etc) to overcome the drawbacks of INL.

## Figures and Tables

**Table 1:** Coded and decoded values of independent and dependent variables for response surface metrology.

Independent variables	Coded	Decoded					Dependent variables	Coded
		- 2	- 1	0	+ 1	+ 2		
MD^1)^ concentration (%, w/v)	X_1_	10	15	20	25	30	Encapsulation yield (%)	Y_1_
Inlet temperature of spray drying (°C)	X_2_	80	88	95	103	110	Viability (%)	Y_2_

^1)^MD: maltodextrin

**Table 2:** Different concentration ratios of wall materials (MD and INL) for encapsulation of LPD2^1)^ through Spray drying.

Wall materials	Concentration Ratio (w/v)
MD10	MD : INL^2)^ = 10 : 0
INL3	MD : INL = 7 : 3
INL5	MD : INL = 5 : 5
INL7	MD : INL = 3 : 7
INL10	MD : INL = 0 : 10

^1)^LPD2: *Lactiplantibacillus plantarum* D-2, ^2)^INL: inulin

**Table 3:** Different combinations of independent and dependent variables for encapsulation of LPD2 with MD through spray drying.

Run no.	Independent variables		Dependent variables	
	X_1_^1)^	X_2_^2)^	Y_1_^3)^	Y_2_^4)^
1	15	103	79.15 ± 1.18^a5)^	94.42 ± 0.84^de^
2	15	88	74.22 ± 0.55^bcd^	95.48 ± 0.23^c^
3	20	80	73.30 ± 2.23^bcde^	96.78 ± 0.34^b^
4	20	95	72.53 ± 1.38^cde^	95.78 ± 1.22^c^
5	20	95	73.09 ± 0.51^bcde^	95.25 ± 0.24^cd^
6	25	103	75.21 ± 0.42^bc^	93.31 ± 0.35^f^
7	30	95	72.27 ± 3.17^de^	93.77 ± 0.15^ef^
8	10	95	78.86 ± 1.58^a^	98.01 ± 0.24^a^
9	20	95	74.91 ± 1.33^bcd^	95.58 ± 0.13^c^
10	25	88	70.73 ± 0.22^e^	95.41 ± 0.11^c^
11	20	95	74.51± 1.13^bcd^	95.56 ± 0.34^c^
12	20	110	75.97 ± 0.34^b^	94.51 ± 0.65^de^
13	20	95	73.56 ± 2.21^bcde^	95.66 ± 0.37^c^

^1)^X_1_ = MD concentration (%), ^2)^X_2_ = Inlet temperature of spray drying (°C), ^3)^Y_1_ = Encapsulation yield (%), ^4)^Y_2_ = Viability of LPD2 (%) after encapsulation, ^5)^mean ± sd (*n* = 3)

^a-e^Means with the same superscript are not significantly different by Duncan’s test (*p* < 0.05)

**Table 4:** ANOVA results of response model equations and the optimum condition for encapsulation of LPD2 with MD through spray drying with predicted and actual values.

Dependent values	Response model equation	*R* ^2^	*p*-value	Lack of fit
Y_1_^1)^	Y_1_ = 117.07 - 20.41X_1_ + 7.30X_1_^2^	0.8366	0.000	0.180
Y_2_^2)^	Y_2_ = 95.30 – 0.80 X_1_ – 0.66X_2_	0.7465	0.046	0.002
Predicted and actual values				
Independent variables	Predicted			
X_1_^3)^	10%			
X_2_^4)^	95°C			
Dependent variables	Predicted		Actual	ARE (%)
Y_1_	79.15		75.34 ± 1.38^5)^	5.05
Y_2_	97.14		92.18 ± 0.48	5.38

^1)^Y_1_ = Encapsulation yield (%), ^2)^Y_2_ = Viability of LPD2 (%) after encapsulation ^3)^X_1_ = MD concentration. ^4)^X_2_ = Inlet temperature (%), ^5)^mean ± sd (*n* = 3)

**Table 5:** Encapsulation yield and viability of LPD2 with different concentration ratios of MD and INL after encapsulation through spray drying.

Wall materials (w/v)	Encapsulation yield (%)	Viability of LPD2 (%)
MD10	75.34 ± 1.38^a1)^	98.80 ± 0.50 ^a^
INL3	76.69 ± 1.64 ^a^	97.45 ± 0.60 ^b^
INL5	75.18 ± 2.44 ^a^	94.62 ± 0.34 ^c^
INL7	75.49 ± 0.87 ^a^	92.27 ± 0.24 ^d^
INL10	74.73 ± 0.62 ^a^	90.08 ± 0.58 ^e^

^1)^mean ± sd (*n* = 3)

^a-e^Means with the same superscript are not significantly different by Duncan’s test (*p* < 0.05)

**Table 6:** Physicochemical characteristics of encapsulated LPD2 with MD10 and INL3.

Wall materials	ρB^1)^	ρt^2)^	HR^3)^	CI (%)^4)^	WSI (%)^5)^	WAI^6)^
MD10	0.47 ± 0.01*^7)^	0.63 ± 0.08	1.32 ± 0.03	24.59 ± 1.72	66.23 ± 3.48	0.63 ± 0.04
INL3	0.59 ± 0.04*	0.72 ± 0.05	1.26 ± 0.12	24.68 ± 1.80	57.08 ± 4.90	0.68 ± 0.06

^1)^ρB = bulk density (g/cm^3^), ^2)^ρt = tap density (g/cm^3^), ^3)^HR = Hausner ratio, ^4)^CI = Carr's index, ^5)^WSI = water solubility index, ^6)^WAI = water absorption index ^7)^mean ± sd (*n* = 3)

*p < 0.05
